# Rhubarb Enema Improved Colon Mucosal Barrier Injury in 5/6 Nephrectomy Rats May Associate With Gut Microbiota Modification

**DOI:** 10.3389/fphar.2020.01092

**Published:** 2020-07-29

**Authors:** Chunlan Ji, Yusheng Deng, Aicheng Yang, Zhaoyu Lu, Yang Chen, Xusheng Liu, Lijuan Han, Chuan Zou

**Affiliations:** ^1^ Second Clinical Medical College, Guangzhou University of Chinese Medicine, Guangzhou, China; ^2^ Department of Nephrology, Guangdong Provincial Hospital of Chinese Medicine, Guangzhou, China; ^3^ Department of Scientific Research, KMHD, Shenzhen, China; ^4^ Department of Nephrology, The Affiliated Jiangmen TCM Hospital of Jinan University, Jiangmen, China; ^5^ State Key Laboratory of Dampness Syndrome of Chinese Medicine, The Second Affiliated Hospital of Guangzhou University of Chinese Medicine, Guangzhou, China

**Keywords:** chronic kidney disease, gut microbiota, intestinal barrier, rhubarb enema, systemic inflammation

## Abstract

Chronic kidney disease (CKD) is often accompanied with colon mucosal barrier damage and gut microbiota disturbance, which strongly associate with up-regulated inflammation and kidney tubulointerstitial fibrosis. However, few interventions could protect the damaged barrier effectively. *Rheum palmatum* L or rhubarb is a common herbal medicine which is widely used to protect the colon mucosal barrier. In previous studies, we found that rhubarb intervention may reduce renal inflammation and tubulointerstitial fibrosis, *via* gut microbiota modification. However, whether intestinal barrier function could be improved by rhubarb intervention and the relationship with intestinal flora are still unknown. Therefore, we investigated the effects of rhubarb enema on intestinal barrier, and further analyzed the relationship with gut microbiota in 5/6 nephrectomy rats. Results indicated that rhubarb enema improved the intestinal barrier, regulated gut microbiota dysbiosis, suppressed systemic inflammation, and alleviated renal fibrosis. More specifically, rhubarb enema treatment inhibited the overgrowth of conditional pathogenic gut bacteria, including *Akkermansia*, *Methanosphaera*, and *Clostridiaceae* in CKD. The modification of gut microbiota with rhubarb intervention displayed significant correlation to intestinal barrier markers, TLR4–MyD88–NF-κB inflammatory response, and systemic inflammation. These results revealed that rhubarb enema could restore intestinal barrier by modifying several functional enteric bacteria, which may further explain the renal protection mechanism of the rhubarb enema.

## Introduction

Chronic kidney disease (CKD) has become a worldwide health problem with an increasing prevalence. In Europe, the adjusted prevalence of CKD stages 1–5 varied from 3.3% to 17.3%, 6.7% in South China, and 18.3% in Southwest China (Bruck et al., 2016). Previous authors have reported that risk factors may accelerate the progression of CKD. These factors include older age, male, diabetes, proteinuria, hypertension (Mcclellan and Flanders, 2003; Taal and Brenner, 2006) and systemic inflammatory levels, among which systemic inflammation induced by intestinal toxins (Anders et al., 2013), is considered as one of the most important risk factors, and has become a research hot spot in recent years. The intestinal toxins, including trimethylamine-N-oxide (TMAO), indoxyl-sulfate (IS), P-cresol (PCS) and lipopolysaccharide (LPS) (Stubbs et al., 2016; Gryp et al., 2017; Liu et al., 2018), are reportedly elevated due to a leaky intestinal barrier following CKD. These intestinal toxins can cause oxidative stress and inflammation.

The human intestinal barrier is composed of mucous, mechanical, and immune barriers. The mechanical barrier is the basic barrier, composed mainly of intestinal epithelial cells and tight connections between cells (Cliffe et al., 2005). The inflammatory state in the colonic wall of animals with CKD has been shown to impair the intestinal epithelial barrier structure and function, which contributes to the translocation of endotoxins, microbial fragments, and other noxious luminal products into circulation (Vaziri et al., 2012; Lau et al., 2015; Vaziri et al., 2016).

Recent studies have shown that intestinal barrier damage is associated with gut microbiota disorders. The proportion of bacteria in the human gastrointestinal tract is maintained within a certain range (Faith et al., 2013), these bacteria form a symbiotic relationship with the human body and participate in human metabolism, immunity, and other physiological activities (Tremaroli and Backhed, 2012). In patients with CKD, the homeostatic balance among gut microbiota is disrupted, with a decrease in the proportion of probiotic bacteria and an increase in that of pathogenic bacteria. Probiotic bacteria include mainly *Lactobacillus* and *Bifidobacteria*, which contain protein machinery for butyrate production, a derivative of short chain fatty acids. Pathogenic bacteria include mainly *Clostridium* *difficile*, *Escherichia coli*, and *Clostridium perfringens*. These bacteria have protein machinery to produce ammonia and intestinal toxins, which can corrode the intestinal barrier (Wong et al., 2014). Therefore, changes in the gut microbiota are closely related to changes in intestinal barrier function, and the intestinal tract is an important target for CKD treatment. However, clinical treatments targeting the intestine, such as intestinal adsorbent and probiotic supplements, are limited and less effective (Manga et al., 2009; Tadao et al., 2009; Iwao et al., 2011).

Rhubarb comprises the dried roots and rhizomes of *Rheum palmatum* L, *Rheum tanguticum* Maxim. ex Balf, or *Rheum officinale* Baill., belonging to Rheum genus and Polygonaceae family. Rhubarb, used in traditional Chinese herbal medicine, is a strong irritant of the gastrointestinal tract upon oral administration since it contains anthraquinones. Enemas, which are administered *via* the anus, can prevent stimulation of the gastrointestinal tract by oral administration, thereby effectively controlling side effects such as nausea, vomiting, and abdominal distension . Hence, rhubarb is widely used as an enema for its laxative properties across China, especially in CKD. Traditionally, 30 g rhubarb was immersed in 300 mL distilled water for 30 min, then decocted twice with water (300 mL and 100 mL) for 40 min. The resultant extractions were mixed and filtered. The filtrates were concentrated to 100 ml, resulting in a 0.3 g/ml crude drug content for the rhubarb enema. Rhubarb granules present another method of use, which is obtained by decocting, filtering, centrifugation, concentration, and drying processes. One gram rhubarb granule is equivalent to 3 g rhubarb. As a traditional Chinese herbal medicine, rhubarb is widely used in sepsis, severe pancreatitis, and other diseases to protect the intestinal barrier (Wang et al., 2017; Xiong et al., 2018). Besides, rhubarb can slow CKD progression by inhibiting tubulointerstitial fibrosis (Zhang et al., 2015; Zhang et al., 2018), which may be related to disrupted intestinal flora regulation in CKD (Han et al., 2017).Our previous study also showed that rhubarb enema could reduce serum uremic toxin and downregulate systemic inflammation and oxidative stress, thereby improving renal interstitial fibrosis (Lu et al., 2015; Zeng et al., 2016). As rhubarb enema acts directly on the intestine, this study was performed to determine whether rhubarb enema could restore intestinal barrier by modifying functional enteric bacteria in 5/6 nephrectomy rats, and further to explain the renal protective mechanism of the therapy.

## Materials and Methods

### Experimental Animals and Medicinal Intervention

This study was performed with 30 male specific pathogen-free (SPF)-grade Sprague–Dawley rats, aged 12 weeks and weighing approximately 180 ± 20 g. The rats were housed in the SPF animal breeding room of the Academy of Chinese Medicine of Guangdong Province and given free access to water. The rats were fed on a 12-h light/dark cycle. This experimental study was approved by the ethics review committee of Guangdong Hospital of Traditional Chinese Medicine (NO., 2018028).

The classic 5/6 nephrectomy (Nx) model was used in this study. Thirty rats were divided randomly into the sham group (n = 10) and CKD group (n = 20) after 5/6 Nx was performed to remove the upper and lower two-thirds of the left kidney. Total right Nx was performed 7 days post-surgery. Rats in the sham group were stripped of the fat sac without Nx. Eight weeks after the operation, rats in the CKD group were divided randomly into the 5/6 Nx model group and the 5/6 Nx + rhubarb enema group.

**Figure 1:**
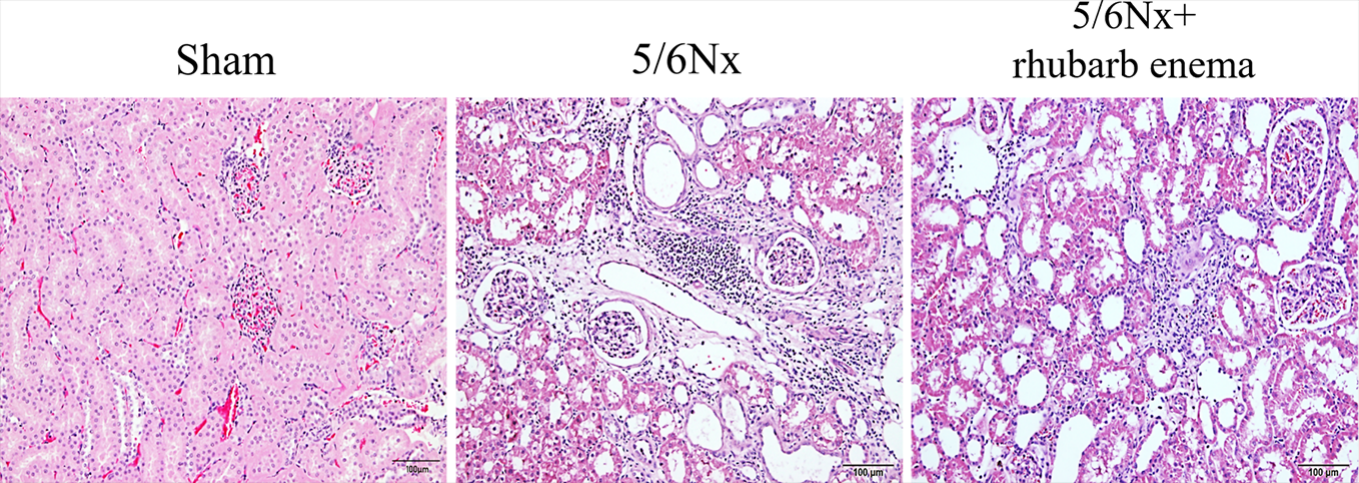
Renal histopathological changes (hematoxylin and eosin (HE) stain). A marked enlargement of the renal tubular lumen, renal tubular atrophy, mononuclear lymphocyte infiltration, and interstitial fibrosis in 5/6Nx compared with Sham group; while these histopathological changes were improved by *rheum palmatum* L enema treatment. Magnifications × 100.

Rhubarb granules (batch number, 19051281) were obtained from Jiangyin Tianjiang Pharmaceutical Industry Co., Ltd. The herbal materials were authenticated according to Chinese Pharmacopeia, 2015, morphologically and chemically. The voucher species were stored at the Center for Chinese Medicine at Guangdong Hospital of Traditional Chinese Medicine. The rhubarb enema water was prepared with 20 g rhubarb granules dissolved in 100ml distilled water. Rhubarb enema was performed with a dose of 2.12 g/kg once per day for 4 weeks, according to clinical administration (Luo et al., 2015). High-performance liquid chromatography–mass spectrometry (HPLC-MS) analysis was conducted to confirm the quality of the rhubarb granules extract (**Supplementary Table 1** and **Figure 1**).

### Detailed Operation Instructions for Enema

The rats were fasted for12 h before enema. The rat was held on the rat frame so that the head and neck were behind, the tail and the anus were presented in front, and the tail of the rat faced upward to expose the anus. The abdomen of the rat was stroked to encourage defecation, ensuring that the intestines were empty. The enema solution was maintained at the same temperature of the rat anus (37.5-39 °C). A straight-head gavage needle was inserted about 8 cm into the rat rectum, since research shows that the rat rectum measures approximately 8 cm, the anus was squeezed, and the needle position was fixed. Then, the drug was slowly injected while the anus was pinched for 30 s to 2 min, to retain the enema solution. The enema was administered once a day, for 4 weeks. The drugs and instruments were treated aseptically during this process.

### UPLC-ESI-MS Analysis

Chromatographic separation was performed on a Dionex UltiMate, 3000 UPLC system (Thermo Scientific, San Jose, CA, USA). UPLC conditions were as follows: Waters acquity SB-C18 column (4.6 × 100 mm, 1.8 μm, Agilent Technologies, CA, USA); column temperature: 30°C; mobile phase: 0.1% formic acid water (A) and acetonitrile (B); gradient conditions for positive mode analysis: 0–4 min 10–13% B; 4–6 min 13–50% B; 6–10 min 50% B; 10–14 min 50–85% B; 14–18 min 85% B; 18–20 min 85–100%; 20–25 min 100%; re-equilibrate: 10 min; gradient conditions for negative mode analysis: 0–2 min 15% B; 2–4 min 15–55% B; 4–8 min 55% B; 8–14 min 55–70% B; 14–22 min 70–80% B; 22–24 min 80–100%; 24–28 min 100%; re-equilibrate: 10 min; flowrate: 0.2 ml/min; injection volume: 2 μl. We tested 6 samples from the same batch and measured the peak area every 2 h to investigate the repeatability and stability of the experiment.

MS detection was performed on an Orbitrap mass spectrometer (Thermo Fisher), which was operated in both positive (ESI+) and negative electrospray ionization interface (ESI−). The MS parameters were as follows: heater temperature, 350°C; sheath gas flowrate, 35 arb; aux gas flowrate, 10 arb; sweep gas flowrate, 0 arb; I spray voltage, 3.2 kV; capillary temperature: 300°C; mass range: m/z 150–1,500. Repeatability and stability were tested as above.

### Biochemical Detection

Serum samples were sent to the Laboratory Department of University City Hospital of Guangdong Hospital of Traditional Chinese Medicine, serum creatinine, blood urea nitrogen, and serum albumin were detected using a Roche biochemical analyzer (Hitachi, 7180, Tokyo, Japan).

### Histopathological Studies

Kidney and colon tissues were dehydrated and embedded, and paraffin specimens were made and sectioned for hematoxylin and eosin (HE) staining. Histological images were captured using a microscope (Hitachi).

### Enzyme-Linked Immunosorbent Assay

The levels of interleukin (IL)-1β, IL-6, and lipopolysaccharide (LPS) in serum and kidney tissues were detected using ELISAs. IL-1β (ab100768, Abcam) and IL-6 (ab100772, Abcam) assay kits were obtained from Abcam (Cambridge, UK). LPS assay kit (OKEH02594) were obtained from AVIVA SYSTEMS BIOLOGY (California, USA).

### Western Blot Analysis

Colon tissues were lysed in 1 mL RIPA lysis buﬀer containing 1 mM PMSF and 1% phosphatase inhibitor cocktail (Applygen Technologies Inc., Beijing, China). Protein concentrations were measured using a Pierce BCA protein assay kit (Thermo Fisher Scientific, Waltham, Massachusetts, USA). Samples (25–50 μg) were separated using 10% SDS–PAGE and then wet transferred to polyvinylidene diﬂuoride membranes (PVDF, EMD Millipore, Burlington, USA). Membranes were blocked with 5% non-fat milk and incubated with primary antibodies against occludin (1:1000; ab167161, Abcam), claudin-1 (1:1000; ab180158, Abcam), ZO1 (1:1000; CST#13663,Cell Signaling Technologies, Darmstadt, Germany), TLR4 (1:500; sc-293072, Santa Cruz), NF-κB (1:1000; CST#8242, Cell Signaling Technologies), phosphorylated (p) NF-κB (1:1000; CST#8214, Cell Signaling Technologies), MyD88 (1:1000; ab2064, Abcam), or β-actin (1:1000; CST#4970, Cell Signaling Technologies) and incubated overnight at 4°C with gentle shaking. The membranes were washed three times and then incubated with a horseradish peroxidase–linked secondary antibody (1:1000; Beyotime, Jiangsu, China). Immunoreactivity was detected using an enhanced chemiluminescence reagent (WBKLS0500; EMD Millipore) and quantiﬁed using Image Lab 5.2.1 software (Bio-Rad, Hercules, CA, USA).

### Stool Specimen Collection

A disposable sterile medical pad was placed on the operating table and the rat was held on the rat frame so that the head and neck were behind, the tail and anus were presented in front, and the tail of the rat faced upward to expose the anus. The abdomen was stroked to promote defecation. Using sterile forceps, fresh feces was collected, placed into EP tubes, and quickly placed in liquid nitrogen. Thereafter, samples were stored at -80°C.

### Genomic DNA Extraction and 16S Amplification

DNA samples were quantified using a NucleoSpin Soil Kit - Macherey-Nagel (Biocompare, Germany) and then transferred to BGI Genomics for gene sequencing of the V4 region of the 16S rRNA gene with the Hiseq, 2500 (Illumina, California, USA). PCR primers used for the 16S rRNA amplicon libraries were 515F and 806R.

### Bioinformatics and Biostatistics

The raw sequencing data were filtered to obtain clean reads by eliminating adapter contamination and low quality. Overlapping paired-end reads were merged to tags using FLASH (version 1.2.11), then clustered to operational taxonomic units (OTUs) at 97% sequence similarity by USEARCH (version 7.0.1090). Taxonomic ranks were assigned to OTU representative sequences using the Ribosomal Database Project Classifier (version 2.2) trained on the database Greengene_2013_5_99, using confidence values of 0.6 as cutoff values. Functional classification schemes of KEGG Orthology were predicted using PICRUSt (version 1.1.3).

All statistical analyses were performed using R software (version 3.5.0; R Foundation for Statistical Computing, Vienna, Austria). Alpha diversity (Shannon index) and principal coordinate analysis (PCoA) based on Bray-Curtis dissimilarity matrices were calculated at the genus level for 16S rDNA gene sequencing using the vegan package. The Kruskal-Wallis test was performed to compare alpha diversity among groups. Permutational multivariate analysis of variance (PERMANOVA) was performed on dissimilarity matrices to assess the effects of groups with 10,000 permutations in R (version 3.5.0, vegan package). Linear discriminant analysis of effect size (LEfSe) was used to identify features that differed significantly among groups, as well as their effect sizes. EnvFit analysis was used to determine the effect size and significance of each covariate among the centroids of each group with 999 permutations using the envfit function; redundancy analysis (RDA) was performed using the rda command of the vegan package. Spearman Rank Correlation was performed to determine correlations between differential microbiota/KO and environmental variables; the false discovery rate was used to assess the significance of differences with *P* < 0.05.

### Statistical Method

SPSS software (version 19.0, IBM Corp, Armonk, NY, USA) was used for statistical analysis, and the measurement data were assessed for adherence to normal distribution. Independent-samples T tests were used to compare normally distributed data with homogeneous variance between two groups. Single-factor analysis of variance was used for multi-group data. The non-parametric Mann-Whitney U test was used to compare non-normally distributed data with heterogeneous variance between two groups. The nonparametric rank-sum test was used for comparison of rank data between groups. *P* values < 0.05 were considered statistically significant.

## Results

### Standardization of Rhubarb Granules

Before drug treatment, rhubarb granules were chemically standardized. An HPLC-MS approach was established to reveal the chemical profile of rhubarb granules and quantify the main ingredients in the extract. Using the respective individual standards, five compounds, including gallic acid, aloe-emodin-β-D-glucopyranoside, emodin-β-d-glucopyranoside, rhein, and emodin, were identified from rhubarb granules (**Supplementary Table 1** and **Figure 1**). The chemical analysis of rhubarb granules here served as quality control for the reproducibility of the below animal study.

### Rhubarb Enema Reduces Blood Creatinine and Improves Renal Fibrosis

At the end of the experiment, the serum creatinine and blood urea nitrogen levels of rats in the 5/6 Nx model and 5/6 Nx + rhubarb enema groups were significantly higher than those of the sham group(*P*<0.001), while the serum creatinine level was significantly lower in the 5/6 Nx + rhubarb enema group than in the 5/6 Nx model group (*P*=0.042) (**Table 1**).

**Table 1:** Physical and Biochemical characteristics.

Parameters	Sham (n=10)	5/6Nx (n=10)	5/6Nx + rhubarb (n=10)
Body weight, g	516.8 ± 35.6	528.3 ± 52.2	523.6 ± 45.3
Cr, umol/L (before)	30.9 ± 2.3	58.1 ± 6.1*	/
Urea, mmol/L (before)	7.24 ± 0.7	13.2 ± 1.4*	/
Cr, umol/L (after)	45.0 ± 7.4	76.3 ± 8.5*	67.1 ± 5.1#
Urea, mmol/L(after)	6.8 ± 1.1	11.0 ± 1.1*	9.9 ± 1.5
Alb, g/L	41.5 ± 2.8	40.3 ± 2.8	40.7 ± 1.4
Urine volume, mL	22.3 ± 10.1	25.0 ± 8.7	26.3 ± 7.3
24h Urine protein, g	0.9 ± 0.6	0.9 ± 0.5	0.9 ± 0.3
ALT/AST	3.9 ± 1.2	4.1 ± 1.2	4.0 ± 0.6

Alb, albumin; Cr, creatinine; ALT, alanine transaminase; AST, aspartate transaminase. *Sham Vs 5/6Nx; #5/6Nx Vs 5/6Nx + rhubarb. Before, before rhubarb enema treatment; after, after rhubarb enema treatment.

In the 5/6 Nx model group, HE staining of renal tissues showed obvious enlargement of the renal tubular lumen, as well as renal tubular atrophy, mononuclear lymphocyte infiltration, and interstitial fibrosis; these histopathological changes were improved by rhubarb enema treatment (**Figure 1**). These results demonstrated that rhubarb enema could improve renal functionality to a certain degree.

### Rhubarb Enema Improved Systemic Inflammation

IL-1β and IL-6 levels in serum and kidney tissues of the 5/6 Nx model and 5/6 Nx + rhubarb enema groups were significantly higher than those of the sham group (*P*<0.01), whereas those of the 5/6 Nx + rhubarb enema group were in between, lower than those of the model group with statistical significant, but higher than those of the sham group. Serum LPS levels in the 5/6 Nx model group and the 5/6 Nx + rhubarb enema group were significantly higher than that of the sham group (*P*<0.01), while that of the 5/6 Nx + rhubarb enema group were in between, significantly lower than that of the 5/6 Nx model group (*P*<0.01), but higher than that of the sham group (**Table 2**). These results suggest that rhubarb enema intervention can potentially reduce systemic inflammation.

**Table 2:** systemic inflammation levels.

Parameters	IL-1β		IL-6		LPS
	Serum	Kidney	Serum	Kidney	Serum
Sham	4.7 ± 2.9	7.4 ± 2.0	35.6 ± 33.7	5.4 ± 2.7	25.9 ± 15.5
Model	29.1 ± 9.9*	14.4 ± 0.9*	459.3 ± 137.2*	16.7 ± 1.8*	109.5 ± 33.3*
rhubarb enema	14.2 ± 3.4^#^	11.9 ± 1.7^#^	218.1 ± 105.7^#^	13.8 ± 2.1^#^	55.6 ± 10.7^#^

LPS, lipopolysaccharide. *Sham Vs 5/6Nx; #5/6Nx Vs 5/6Nx + rhubarb enema.

### Rhubarb Enema Improved Intestinal Barrier Integrity

**Figure 2:**
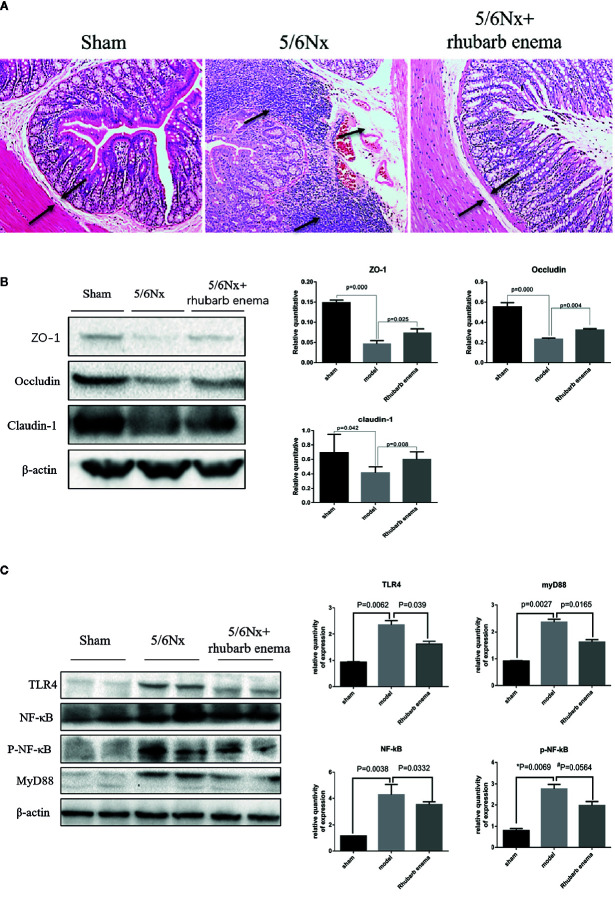
Rhubarb enema improved the intestinal barrier. **(A)** Colon histopathological changes (hematoxylin and eosin (HE) stain). Compared with the sham group, the lamina propria and mucosal layer of the colon were obvious oedematous; moreover, obvious infiltration of lymphocyte monocytes in the mucosal layer was observed in the 5/6Nx model group. Following rhubarb enema, the edema in the lamina propria and mucosal layer was significantly improved, and the infiltration of inflammatory cells in the mucosal layer was reduced. Magnifications × 100. **(B)** The intestinal mucosal barrier (ZO1, occludin, and claudin-1) in colon tissues was significantly lower in the 5/6Nx model rat; this expression was significantly higher in the 5/6Nx + rhubarb enema group than in the 5/6Nx model group. **(C)**TLR4 signaling pathways is activated in in the colon tissue of 5/6Nx model rat while significantly reduced by rhubarb enema treatment.

In the 5/6Nx model group, HE staining of colon tissue showed obvious edema in the lamina propria and mucosal layer of the colon compared with the sham group. Moreover, obvious infiltration of lymphocytes and monocytes in the mucosal layer was observed in the 5/6 Nx model group. Following rhubarb enema, the edema in the lamina propria and mucosal layer was significantly improved, and the infiltration of inflammatory cells in the mucosal layer was reduced (**Figure 2A**).

The expression of key indicators of the intestinal mucosal barrier (occludin, claudin-1, and ZO1) in colon tissues was significantly lower in the 5/6 Nx model and 5/6 Nx + rhubarb enema groups than the sham group (*P*<0.01; *P* =0.042*; P*<0.01); this expression was significantly higher in the 5/6 Nx + rhubarb enema group than in the 5/6 Nx model group (*P*<0.01; *P*<0.01; *P* =0.025, **Figure 2B**).

TLR4 signaling pathways have been shown to be activated in the colon tissue of CKD model rats. In this study, the expression levels of TLR4, NF-κB, pNF-κB, and MyD88 in the colon tissue were significantly higher in the 5/6Nx model and 5/6Nx + rhubarb enema groups than in the sham group (*P* < 0.01, respectively). Rhubarb enema treatment significantly reduced expression levels of TLR4, NF-κB, pNF-κB, and MyD88 in the colon tissue relative to those of the 5/6Nx model group, (*P* = 0.039; *P* = 0.033; P =0.056; *P* = 0.016, **Figure 2C**).These results indicate that rhubarb enema improved the intestinal barrier upon 5/6 Nx.

### Changes to Gut Microbiota After Rhubarb Enema Treatment

#### Diversity Analysis

**Figure 3:**
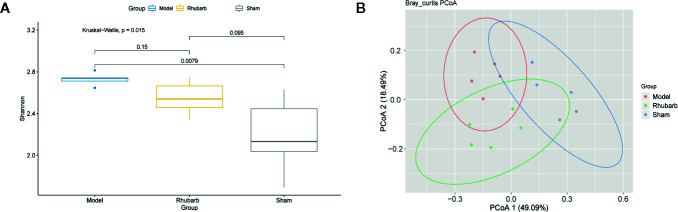
Rhubarb enema treatment modify the diversity of gut microbiota. The diversity of gut microbiota was significantly different among three groups. **(A)** The Shannon index of the model group was higher than the rhubarb enema group, and significantly higher than the sham group. **(B)** The principal component analysis (PCoA) plots were based on Bray-Curtis distance of the genus levels for 16S rDNA gene sequencing. Individuals (represented by points) were clustered into 3 groups. Permutational multivariate analysis of variance (PERMANOVA) was performed on dissimilarity matrices to assess the effects of groups with 10,000 permutations.

Alpha diversity represents the taxonomy richness and unevenness of a micro-ecology. Here we evaluated alpha diversity within each sample and each group by calculating the Shannon index at the genus level. The Shannon index differed significantly among the three groups (*P* = 0.015, Kruskal-Wallis test, **Figure 3A**). It was significantly higher in the 5/6 Nx model group than in the sham group (*P* = 0.0079). Interestingly, the diversity of the rhubarb enema group was lower than that of the 5/6 Nx model group, but higher than that of the sham group. It seems that rhubarb enema tended to correct the disease tendency.

We evaluated the beta diversity of the samples using PCoA based on Bray-Curtis distance to investigate the difference of microbiota composition among groups. Individuals were clustered into three clusters (**Figure 3B**). Interestingly, the individuals in the same group form a cluster, the inter-group distance was significantly greater than the intra-group distance (*P* < 0.001, 9999 permutations).

#### Differential Abundance Analysis

**Figure 4:**
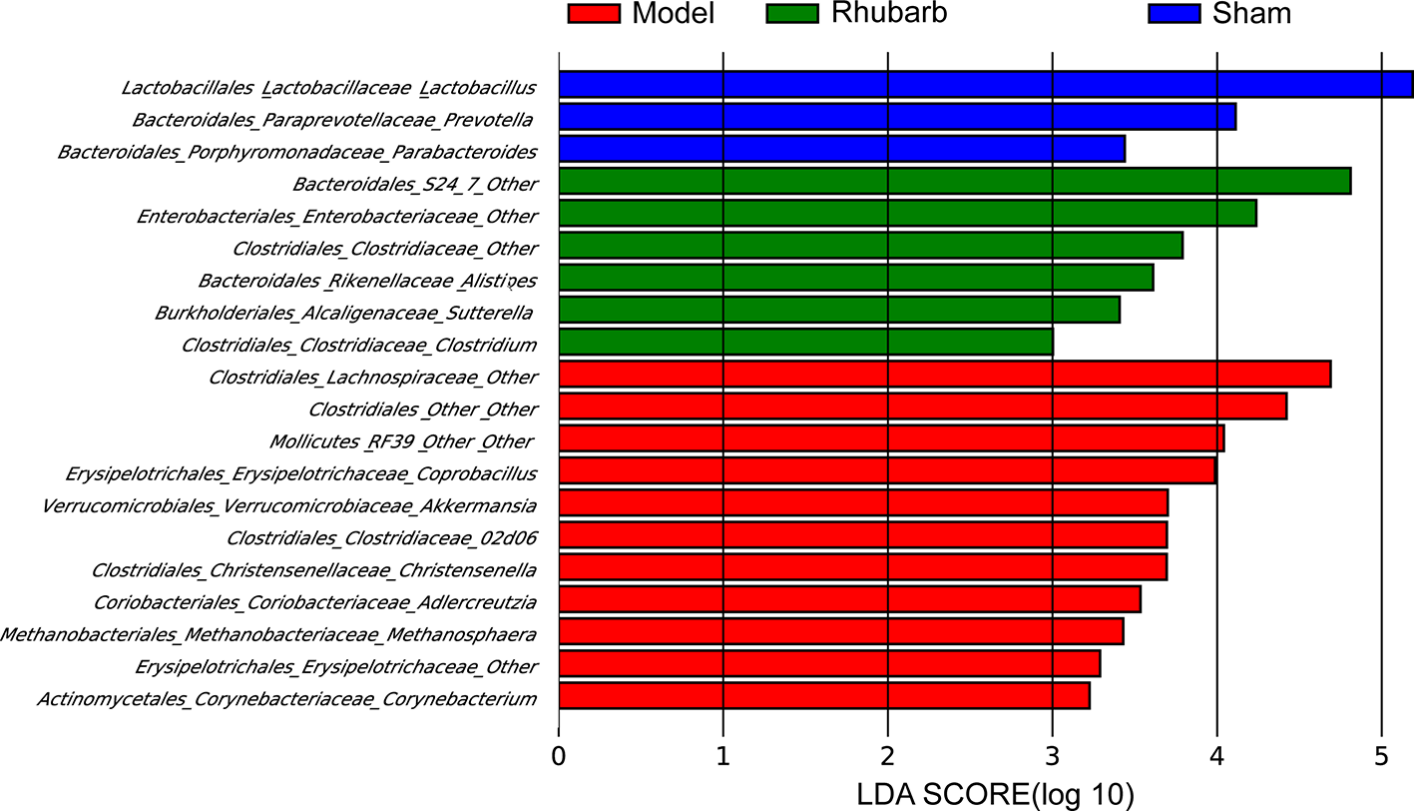
Rhubarb enema treatment modify gut microbiota that enriched in different group. LEfSe was further used to analyze differences gut microbial enriched in the three groups.

LEfSe analysis was applied to identify differentially abundant bacterial taxa among the three groups, only those taxa that obtain a log LDA score >2 are ultimately considered. Genera with higher abundance in the sham group included *Lactobacillus*, *Prevotella*, and *Parabacteroides*. Whereas *Methanosphaera*, *Akkermansia*, *Christensenella*, *Adlercreutzia*, *Corynebacterium*, *Coprobacillus*, *RF39* from class *Mollicutes*, *02d06* from family *Clostridiaceae*, one member of family *Erysipelotrichaceae* and two members of order *Clostridiales* (*Lachnospiraceae* and other) were more abundant in the 5/6 Nx group. *Clostridium*, *Alistipes*, *Sutterella*, *S24−7* from order *Bacteroidales*, one member from family *Clostridiaceae*, and one member from family *Enterobacteriaceae* were more abundant in the 5/6 Nx + rhubarb enema group (**Figure 4**).

#### Impact of Environmental Factors on Microbiota Composition

**Figure 5:**
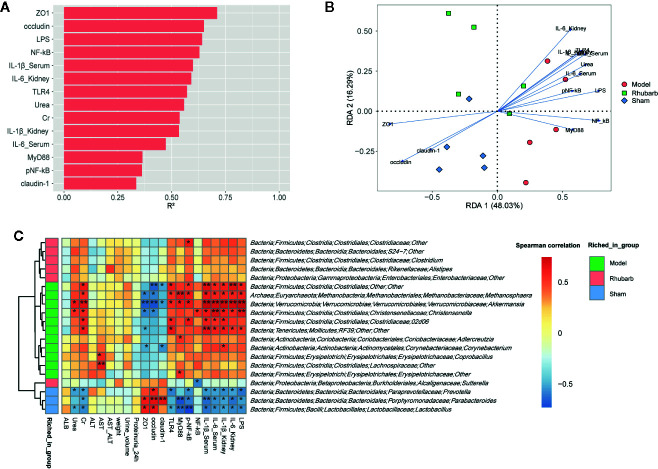
The relationships among the gut microbiota, intestinal barrier, and systemic inflammation. Envfit function was applied to identify the impact of environmental variables on the microbiota variation **(A)**. RDA showed that gut microbiota was correlated with systemic inflammation and intestinal barrier indicators in the three groups **(B)**. Spearman rank correlation between differential gut microbiota and intestinal barrier, systemic inflammation **(C)**.

Envfit function was applied to identify the impact of environmental variables on microbiota variation (**Figure 5A** and **Supplementary Table 4**, P < 0.1, 999 permutations). ZO1, occludin, LPS, and NF-κB exerted the greatest impact on the microbial composition (R2 > 0.6, P < 0.01). RDA biplot was used to present the correlation and the impact of environmental variables on the microbiota variation. It revealed that the expression levels of intestinal barrier tight junction proteins (ZO1, occludin, and claudin-1) were positively correlated with each other, and negatively correlated with systematic inflammatory cytokines (IL-6, IL-1β), key proteins of the TLR4 pathway (TLR4, NF-κB, pNF-κB, and MyD88), and LPS. Systematic inflammatory cytokines were positively correlated with key proteins of the TLR4 pathway and LPS. Moreover, ZO1, occludin, and claudin-1 were found to contribute to the microbial variation of the sham group. Systematic inflammatory cytokines, key proteins of the TLR4 pathway and LPS contributed to the microbial variation of the 5/6 Nx group. The 5/6 Nx + rhubarb enema group was located between the 5/6 Nx group and the sham group. It indicated that the contribution of systematic inflammatory cytokines, key proteins of the TLR4 pathway, and LPS gradually decreases, while that of the intestinal barrier tight junction proteins gradually increases microbial variation, upon rhubarb enema treatment (**Figure 5B**).

Furthermore, spearman’s rank correlation was performed to determine correlations between differential microbiota and environmental variables. Genus *Lactobacillus*, *Prevotella*, and *Parabacteroides*, which were enriched in the sham group, were positively correlated with expression levels of intestinal barrier tight junction proteins, and negatively correlated with systematic inflammatory cytokines, key proteins of the TLR4 pathway, and LPS. The results of order *Clostridiales*, genus *Methanosphaera*, *Akkermansia*, *Christensenella*, *02d06* from family *Clostridiaceae*, and *RF39* from class *Mollicutes*, which were enriched in 5/6 Nx group, were reversed. The microbiota enriched in 5/6 Nx + rhubarb enema group were almost independent of environmental variables.

#### Differential Function Analysis

**Figure 6:**
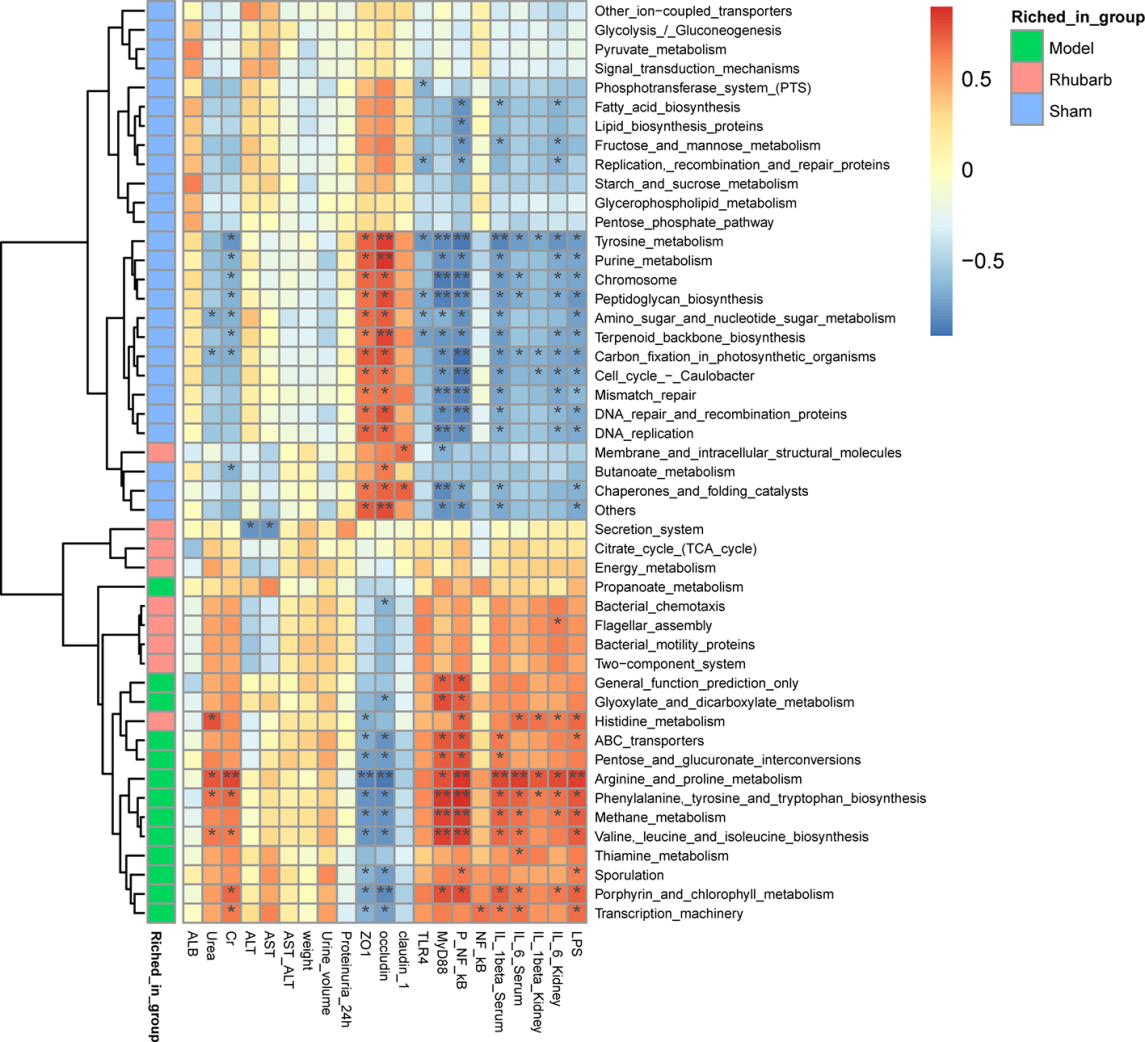
Differential microbiota functional KOs enriched in different groups and the correlated relationship among the gut microbiota functional Kos, intestinal barrier, and systemic inflammation.

Spearman’s rank correlation was also performed to determine correlations between differential KOs and environmental variables (**Figure 5C**). Similar to the result of differential microbiota, functional KOs enriched in the 5/6 Nx group were negatively correlated with the expression levels of intestinal barrier tight junction proteins, and positively correlated with systematic inflammatory cytokines, key proteins of the TLR4 pathway, and LPS, especially arginine and proline metabolism. Whereas the result of KOs enriched in the sham group were reversed, such as tyrosine metabolism (**Figure 6**).

## Discussion

Clinical and experimental studies have shown that intestinal barrier defects have been associated with a wide array of diseases, including both enteral disorders such as irritable bowel syndrome (IBS) and inflammatory bowel disease (IBD) (Brandtzaeg, 2011; Michael et al., 2012; Salim and Söderholm, 2015), and extra-intestinal disorders such as nonalcoholic fatty liver disease, pancreatitis, and obesity (Luca et al., 2009; Yan et al., 2012; Xiong et al., 2018). The latest study found that intestinal barrier function decreased in CKD as well (Alice et al., 2015; Briskey et al., 2016; Meijers et al., 2018). Since the glomerular filtration rate is dramatically reduced in CKD (Wong et al., 2014), the heavy influx of urea from body fluids to the gastrointestinal tract, was firstly hydrolyzed to ammonia and then converted to ammonium hydroxide (NH4OH), leading to increased intestinal pH. The alternated intestinal milieu together with the accumulation of uremic toxins would damage intestinal tight junction, resulting in the activation of submucosal inflammation and macrophage TLR4–MyD88–NF-κB signaling pathways (Williams et al., 2013; Ramezani and Raj, 2014; González-Guerrero et al., 2017). A large number of inflammatory mediators are then produced (Qian, 2017), increasing damage to intestinal epithelial cells (Ali and Raj, 2013), causing enterogenous harmful metabolites and endotoxins to enter systemic circulation, stimulating the onset of systemic inflammation (Ramezani et al., 2016; Vaziri et al., 2016; Briskey et al., 2017). Systemic inflammation is an important risk factor for renal fibrosis (Lau et al., 2015).

Intestinal barrier is affected by many factors, such as environmental factors, including smoking, diet, use of antibiotics, and hyperoxia (Chou and Chen, 2017; Metidji et al., 2018) and especially gut microbiota (Wang H. et al., 2014). CKD is often accompanied by gut microbiome alternation in diversity, quantity, structure, function, and performance (Jiang et al., 2016). According to a previous gut microbiota study of CKD stage 4 and 5 patients, renal failure accompanied an increase in gut microbiota diversity (Feng et al., 2019), probably resulting from the overgrowth of pathogenic bacteria. With the decrease of probiotic bacteria in CKD, protective factors, such as SCFAs produced by enterobacteria *Lactobacillus* and *Prevotellaceae* were also decreased. SCFAs play an important role in maintaining the intestinal barrier (Kelly et al., 2015; Feng et al., 2018). While pathogenic bacteria significantly increased in CKD, such as *Alteromonadaceae*, *Cellulomonadaceae*, *Clostridiaceae*, *E. coli*, and *Enterobacteriaceae*, pathogenic factors such as circulating endotoxins and ammonia produced by these bacteria increased, followed by increased damage to the intestinal barrier (Walker and Lawley, 2013; Wong J et al., 2014).

Effective improvement of the intestinal environment is the key to alleviate CKD progression, but current therapies are limited, such as intestinal adsorbent, probiotic supplements (Manga et al., 2009; Tadao et al., 2009; Iwao et al., 2011). Antibiotics or prebiotics are used to dysregulate the dysbiosis of gut microbiome in kidney disease, thereby alleviating kidney disease (Chen Y.-Y. et al., 2019). Probiotics or antibiotics intervene in the intestinal flora of CKD to reduce harmful products produced by the gut microbiome, such as IS and PCS, which induces systemic inflammation and cardiovascular events in CKD (Li et al., 2015). The application of probiotics or antibiotics can regulate the mucosa and reduce Harmful IgA1 production, thereby delaying the progress of IgAN (Chemouny et al., 2018). The application of probiotics or antibiotics can also reduce proteinuria and gut microbiome-derived phenyl sulfate (Kikuchi et al., 2019).

Rhubarb is a well-known Chinese herbal medicine used to treat various diseases for hundreds of years in China. Researches have shown that rhubarb can treat many diseases by improving intestinal barrier function. For example, rhubarb can improve the intestinal barrier and reduce translocation of bacteria in sepsis (Wang et al., 2017). Rhubarb could protect against intestinal injury and improve intestinal mucosal barrier function in acute pancreatitis (Xiong et al., 2018), as well as in hepatic inflammation (Neyrinck et al., 2017). Studies have shown that rhubarb can regulate the gut microbiota and reduce the inflammatory response to treat type 2 diabetes, with observed increases in the probiotic *Lactobacillus* and short-chain fatty acid-producing bacteria, and decreases in the *Lachnospiraceae* NK4A136 group and LPS-producing Desulfovibrio (Cui et al., 2019).

Rhubarb is commonly used to treat kidney diseases in traditional Chinese medicine (Huang et al., 2018). Studies indicated that rhubarb or its extracts could alleviate renal fibrosis (Dou et al., 2019) and rebalance metabolic dysfunction in diabetic nephropathy (Zhang et al., 2018). Recent studies have shown that there are many other natural products that can alleviate CKD progression by regulating RAS, Smad2/3-TGFβRI, TGF-β/Smad signaling pathway, and the Wnt/β-catenin pathway (Chen et al., 2018a; Chen et al., 2018b).

Our previous study indicated that rhubarb and emodin *via* colonic irrigation, improved systemic inflammation and kidney damage, altered levels of urea and indoxyl sulfate (IS), and changed gut microbiota in rats with CKD (Zeng et al., 2016), and we have explored the dose-effect relationship of rhubarb enema, the optimal dose were selected in this study. Our present study also showed intestinal barrier injury, such as infiltration of lymphocytes and monocytes in the intestinal mucosal layer, activation of the inflammatory signaling pathway in colon tissues, decrease of intestinal tight junction proteins, and significant changes in gut microbiota of 5/6 Nx model rats. Certain probiotic gut microbiota, such as *Lactobacillus*, *Prevotellaceae* and *Parabacteroides* were decreased, whereas conditional pathogenic bacteria, such as *Methanosphaera*, *Akkermansia*, *Christensenella*, *Adlercreutzia*, *Corynebacterium*, *Coprobacillus*, *RF39* from class *Mollicutes*, *02d06* from family *Clostridiaceae*, one member of family *Erysipelotrichaceae* and two members of order *Clostridiales* were increased. Rhubarb treatment significantly inhibited overgrowth of pathogenic gut bacteria, including *Alcaligenaceae*, *Methanosphaera*, and *Clostridiaceae*.

According to researches, the anthraquinones contained in rhubarb have potential nephrotoxicity, hepatotoxicity and intestinal toxicity, such as liver fibrosis (Wang et al., 2011; Wang Y. et al., 2014), apoptosis of human renal tubular epithelial cells (Zeng L.-N. et al., 2013; Cao et al., 2019), and colorectal melanosis or pseudo-melanoderma of colon (Zhu, 2004). All the side effects mentioned above occurred in the case that rhubarb were administered orally for a long medication or overdose. In this study, safe dose of rhubarb was administered in reasonable period *via* anal enema. Anthraquinones are insoluble in water, so anthraquinones administered by enema are hardly absorbed by the intestine, thus the side effects of anthraquinones on the liver, kidneys and other organs are avoided. Until now, there are no reports of toxic effects, such as hepatotoxicity, nephrotoxicity and intestinal toxicity caused by anthraquinones enema both in clinical and animal experiments. On the contrary, in our previous and present studies, rhubarb had a protective effect on the kidney and colon, and can reduced systemic inflammation as well. No toxic reactions were observed in organs such as heart, spleen and liver. In the future, we will pay more attention to the toxicity caused by anthraquinone enema, if necessary.

In our work, UPLC was used to separate methanol pretreated samples, and a total ion chromatogram spectrum was obtained, and the peak was compared with molecular formula data and relevant literatures (Yang et al., 1998; Matsuda et al., 2000; Sun H. et al., 2009; Zheng Q.-X. et al., 2013; Zhao et al., 2018; Wang et al., 2020.) to identify the structure and composition of rhubarb granules, and 5 kinds of the most studied compounds in rhubarb were identified (**Supplementary Table 1**). Our research results are practically consistent with previous research results.

The 5/6 Nx model is one of the classic models to study CKD. Other studies utilizing the 5/6 Nx model have shown that serum creatinine, urea, and proteinuria increased significantly, accompanied by intestinal barrier disruption, dysregulated mucosal Immunity, and decreased expression of HSP70 and claudin-1 in the colon, with increased pore-forming claudin-2 expression and apoptosis, promoting systemic inflammation and renal fibrosis (Yang et al., 2018; Kanemitsu et al., 2019).

The bacteria enriched in 5/6 Nx model rats mainly include *Proteobacteria*, *Betaproteobacteria*, *Enterobacteriaceae*, *Sutterellaceae*, *Clostridiaceae*, which were similar to our findings. These bacteria are related to the metabolism of lipids, amino acids, bile acids, and polyamines. These metabolites are related to CKD risk factors, such as hypertension, inflammation, and fibrosis, caused by LPS leakage. Probiotics and butyric acid can assist in improving the environment by reducing systemic inflammation and renal fibrosis (Kanemitsu et al., 2019).

Dysbiosis of the gut microbiome in UUO model rats shows that *Turicibacter*, *Acetatifactor*, *Blautia*, *Intestinimonas*, and *Oscillibacter* were enriched, with a significant difference in the metabolism of lipids, amino acids, and bile acids metabolism in plasma, which is consistent with the metabolism of intestinal flora related to renal fibrosis, intestinal barrier integrity and systemic inflammation (Chen L. et al., 2019). Oxalate-induced CKD also showed dysbiosis and intestinal barrier dysfunction. Although the restoration of the oxalate-free diet improved renal function to a certain extent, systemic inflammation and renal fibrosis could not be restored (Konrad et al., 2019). In mice with kidney disease under sterile conditions, intestinal levels of indole-3-acetic acid, short-chain fatty acids and n-3 polyunsaturated fatty acids, which play an important role in endothelial barrier function, are significantly reduced. These metabolites originate from the flora. Metabolites may provide renal protection against renal fibrosis by inhibiting destruction of the epithelial barrier (Kanemitsu et al., 2019).

By comparing the present study with others, our results agree with other studies, indicating that rhubarb can reduce pathogenic bacteria in CKD rats, such as Lachnospiraceae and Clostridiaceae, and correct the probiotic deficiency.

In addition, functional correlation results indicated that functional KOs of gut microbiota emerged in three groups were consistent with the above results, and the correlation with inflammation was decreased, while the correlation with barrier protection was enhanced with rhubarb interventions. Researchers found that fatty acid synthase, KOs which emerged in the sham group in our research, helps maintain the mucus barrier by regulating mucin 2, the dominant mucin in the colon and a central component of mucus (Wei et al., 2012), while butanoate can protect epithelial barrier function by preventing the increase of paracellular permeability (Martin-Venegas et al., 2013). L-arginine metabolites, KOs which emerged in the 5/6 Nx model group in our research, may participate in the pathogenesis of kidney disease (Popolo et al., 2014). And tyrosine is digested by intestinal flora to produce phenol and p-cresol (Chang et al., 2017), which can cause system inflammation and CKD progression.

Although the functional genes of related gut microbiota and its KOs responsible for intestinal barrier damage or kidney fibrosis have not been identified very well in the present study, we made some other interesting discoveries. *Clostridium* and t*ryptophan synthesis* were positively correlated with inflammation and negatively correlated with the intestinal barrier. Moreover, *Clostridium* was correlated with urea nitrogen and IS in our previous studies (Zeng et al., 2016). Therefore, we speculated that *Clostridium* contained genes related to enzymes that decompose urea to synthesize ammonia, and genes related to the synthesis of IS by decomposition of tryptophan.

In addition, we did not use a positive drug in this study, because our previous studies have shown that rhubarb enema can significantly improve renal fibrosis and system inflammation in CKD rats, accompanied by a decreased creatinine levels in blood, which may related to the intestinal flora modification by rhubarb (Lu et al., 2015; Zeng et al., 2016). This is not an accidental result, since we conducted a dose-effect relationship exploration between rhubarb enema against renal fibrosis in CKD rats. The optimal dose was applied to this study. We have also tried using sevelamer hydrochloride as a positive control drug to improve renal fibrosis in CKD rats. As an adsorbent, sevelamer can decrease the toxins’ lever in the intestine without being absorbed, thereby reducing system inflammation and improving kidney fibrosis (Sun P. P. et al., 2009; Hauser et al., 2010), which is similar to the role of rhubarb. The preliminary results showed that the effect of rhubarb enema was better than that of sevelamer hydrochloride group. According to recent reports, CKD is usually accompanied by intestinal flora imbalance and intestinal barrier damage, they can cause enteric inflammation, which in turn causes kidney inflammation and renal fibrosis. We have clearly demonstrated that rhubarb enema can improve renal fibrosis. However, whether intestinal barrier function could be improved by rhubarb intervention and the relationship with intestinal flora are still unknown. So we focused investigated the effects of rhubarb enema on intestinal barrier, and further analysis the relationship with gut microbiota in 5/6 nephrectomy rats, and to explain the renal protective mechanism of rhubarb enema.

Taken together, our study partly confirms the hypothesis that rhubarb enema protects the kidney fibrosis by down-regulating systemic inflammation and improving the intestinal barrier in CKD, which may be related to the regulation of related gut microbiota. However, exactly which microflora is related to the barriers has not been clearly proven. In future, studies on metagenomics and flora transplantation are needed to further confirm our hypothesis.

### Limitations

Our results suggest that rhubarb enema significantly improved the intestinal barrier integrity in CKD, the mechanism may be related to the improvement of gut microbiota that regulates the intestinal barrier. Thus far, there are few drugs that can directly regulate the intestinal barrier. However, the correlation between flora and the intestinal barrier in the results of this study was based on bioinformatics analysis, and no relevant experimental verification has been performed.

### Conclusions

The mechanism of intestinal barrier integrity reduction in CKD may relate to intestinal bacteria dysbiosis, and rhubarb enema might regulate the related gut microbiota to down-regulate pathogenic bacteria, regulate intestinal TLR4 signalling pathway, improve intestinal mucosal barrier.

## Data Availability Statement

The data generated for this study can be found in NCBI using accession number PRJNA593972.

## Ethics Statement

This experimental study was approved by the ethics review committee of Guangdong Hospital of Traditional Chinese Medicine (NO., 2018028).

## Author Contributions

The conception and design were proposed by CZ. Animal and molecular biology experiments were mainly performed by CJ. 16S rDNA sequencing and data analysis were performed by YD and LH. The manuscript was drafted by CJ and reviewed by CZ. All authors contributed to the article and approved the submitted version.

## Funding

This study was supported by the National Natural Science Foundation of China (81873142, secured by CZ), Natural Science Foundation of Guangdong Province (2019A1515011054, secured by CZ), the Speciﬁc Foundation of Guangdong Hospital of Chinese Medicine (YN2016MJ05, secured by CZ), and the MOST/SATCM of the People’s Republic of China grant (2013BAI02B04, secured by XL).

## Conflict of Interest

Authors YC and LH were employed by the company KMHD.

The remaining authors declare that the research was conducted in the absence of any commercial or financial relationships that could be construed as a potential conflict of interest.

## References

[B1] Ali R. Raj D. S. (2013). The gut microbiome, kidney disease, and targeted interventions. J. Am. Soc. Nephrol. 25 (4), 657–670. 10.1681/ASN.2013080905 24231662 PMC3968507

[B2] Alice S. Giuseppe R. Irene B. Aderville C. Santo M. Enrico F. (2015). Alterations of intestinal barrier and microbiota in chronic kidney disease. Nephrol. Dial. Transpl. 30 (6), 924–933. 10.1093/ndt/gfu287 25190600

[B3] Anders H. J. Andersen K. Stecher B. (2013). The intestinal microbiota, a leaky gut, and abnormal immunity in kidney disease. Kidney Int. 83 (6), 1010–1016. 10.1038/ki.2012.440 23325079

[B4] Brandtzaeg P. (2011). The gut as communicator between environment and host: Immunological consequences. Eur. J. Pharmacol. 668 (9), S16–S32. 10.1016/j.ejphar.2011.07.006 21816150

[B5] Briskey D. Tucker P. S. Johnson D. W. Coombes J. S. (2016). Microbiota and the nitrogen cycle: Implications in the development and progression of CVD and CKD. Nitric. Oxide 57, 64–70. 10.1016/j.niox.2016.05.002 27164294

[B6] Briskey D. Tucker P. Johnson D. W. Coombes J. S. (2017). The role of the gastrointestinal tract and microbiota on uremic toxins and chronic kidney disease development. Clin. Exp. Nephrol. 21 (1), 7–15. 10.1007/s10157-016-1255-y 26965149

[B7] Bruck K. Stel V. S. Gambaro G. Hallan S. Volzke H. Arnlov J. (2016). CKD Prevalence varies across the european general population. 27 (7), 2135–2147. 10.1681/ASN.2015050542 PMC4926978 26701975

[B8] Cao C. Hui L. Li C. Yang Y. Zhang J. Liu T. (2019). In Vitro Study of the Nephrotoxicity of Total Dahuang (Radix Et Rhizoma Rhei Palmati) Anthraquinones and Emodin in Monolayer Human Proximal Tubular Epithelial Cells Cultured in a Transwell Chamber. J. Tradit Chin Med. 39 (5), 609–623. 32186110

[B9] Chang C. J. Lin C. S. Lu C. C. Martel J. Ko Y. F. Ojcius D. M. (2017). Corrigendum: Ganoderma lucidum reduces obesity in mice by modulating the composition of the gut microbiota. Nat. Commun. 6, 74–89. 10.1038/ncomms16130 PMC5508223 28695905

[B10] Chemouny J. M. Gleeson P. J. Abbad L. Lauriero G. Monteiro R. C. (2018). Modulation of the microbiota by oral antibiotics treats immunoglobulin A nephropathy in humanized mice. Nephrol. Dial. Transpl. 34 (7), 1135–1144. 10.1093/ndt/gfy323 30462346

[B11] Chen Y.-Y. Chen D.-Q. Chen L. Liu J.-R. Vaziri N. D. Guo Y. (2019). Microbiome-metabolome reveals the contribution of gut-kidney axis on kidney disease. J. Transl. Med. 17 (1), 5. 10.1186/s12967-018-1756-4 30602367 PMC6317198

[B12] Chen D. Q. Hu H. H. Wang Y. N. Feng Y. L. Cao G. Zhao Y. Y. (2018a). Natural products for the prevention and treatment of kidney disease. Phytomedicine 50, 50–60. 10.1016/j.phymed.2018.09.182 30466992

[B13] Chen D. Q. Feng Y. L. Cao G. Zhao Y. Y. (2018b). Natural Products as a Source for Antifibrosis Therapy. Trends Pharmacol. Sci. 39 (11), 937–952. 10.1016/j.tips.2018.09.002 30268571

[B14] Chen L. Chen D.-Q. Liu J.-R. Zhang J. Vaziri N. D. Zhuang S. (2019). Unilateral ureteral obstruction causes gut microbial dysbiosis and metabolome disorders contributing to tubulointerstitial fibrosis. Exp. Mol. Med. 51 (3), 38. 10.1038/s12276-019-0234-2 PMC6437207 30918245

[B15] Chou H. C. Chen C. M. (2017). Neonatal hyperoxia disrupts the intestinal barrier and impairs intestinal function in rats. Exp. Mol. Pathol. 102 (3), 415–421. 10.1016/j.yexmp.2017.05.006 28506763

[B16] Cliffe L. J. Humphreys N. E. Lane T. E. Potten C. S. Cath B. Grencis R. K. (2005). Accelerated intestinal epithelial cell turnover: a new mechanism of parasite expulsion. Science 308 (5727), 1463–1465. 10.1126/science.1108661 15933199

[B17] Cui H. X. Zhang L. S. Luo Y. Yuan K. Huang Z. Y. Guo Y. (2019). A Purified Anthraquinone-Glycoside Preparation From Rhubarb Ameliorates Type 2 Diabetes Mellitus by Modulating the Gut Microbiota and Reducing Inflammation. Front. Microbiol. 10, 1423. 10.3389/fmicb.2019.01423 31293553 PMC6603233

[B18] Dou F. Liu Y. Liu L. Wang J. Sun T. Mu F. (2019). Aloe-Emodin Ameliorates Renal Fibrosis Via Inhibiting PI3K/Akt/mTOR Signaling Pathway *In Vivo* and *In Vitro* . Rejuvenation Res. 22 (3), 218–229. 10.1089/rej.2018.2104 30215298

[B19] Faith J. J. Guruge J. L. Charbonneau M. Subramanian S. Seedorf H. Goodman A. L. (2013). The long-term stability of the human gut microbiota. Science 341 (6141), 1237439. 10.1126/science.1237439 23828941 PMC3791589

[B20] Feng Y. Wang Y. Wang P. Huang Y. Wang F. (2018). Short-Chain Fatty Acids Manifest Stimulative and Protective Effects on Intestinal Barrier Function Through the Inhibition of NLRP3 Inflammasome and Autophagy. Cell Physiol. Biochem. 49 (1), 190–205. 10.1159/000492853 30138914

[B21] Feng Y.-L. Cao G. Chen D.-Q. Vaziri N. D. Chen L. Zhang J. (2019). Microbiome–metabolomics reveals gut microbiota associated with glycine-conjugated metabolites and polyamine metabolism in chronic kidney disease. Cell Mol. Life Sci. 76 (24), 4961–4978. 10.1007/s00018-019-03155-9 31147751 PMC11105293

[B22] González-Guerrero C. Cannata-Ortiz P. Guerri C. Egido J. Ortiz A. Ramos A. M. (2017). TLR4-mediated inflammation is a key pathogenic event leading to kidney damage and fibrosis in cyclosporine nephrotoxicity. Arch. Toxicol. 91 (4), 1925–1939. 10.1007/s00204-016-1830-8 27585667

[B23] Gryp T. Vanholder R. Vaneechoutte M. Glorieux G. (2017). p-Cresyl Sulfate. Toxins 9 (2), 52. 10.3390/toxins9020052 PMC5331431 28146081

[B24] Han W. B. Liu Y. L. Wan Y. G. Sun W. Tu Y. Yang J. J. (2017). Pathomechanism and treatment of gut microbiota dysbiosis in chronic kidney disease and interventional effects of Chinese herbal medicine. Zhongguo Zhong Yao Za Zhi. 42 (13), 2425–2432. 10.19540/j.cnki.cjcmm.20170609.014 28840678

[B25] Hauser A. B. Azevedo I. R. F. Gonçalves S. Stinghen A. Aita C. Pecoits-Filho R. (2010). Sevelamer Carbonate Reduces Inflammation and Endotoxemia in an Animal Model of Uremia. Blood Purif. 30 (3), 153–158. 10.1159/000319850 20861617

[B26] Huang K. C. Su Y. C. Sun M. F. Huang S. T. (2018). Chinese herbal medicine improves the long-term survival rate of patients with chronic kidney disease in Taiwan: a nationwide retrospective population-based cohort study. Front. Pharmacol. 9, 1117. 10.3389/fphar.2018.01117 30327604 PMC6174207

[B27] Iwao N. Motonobu N. Koji K. Toshihisa O. Ikuo K. Kazumi U. (2011). Effects of synbiotic treatment on serum level of p-cresol in haemodialysis patients: a preliminary study. Nephrol. Dial Transpl. 26 (3), 1094–1098. 10.1093/ndt/gfq624 20929916

[B28] Jiang S. Xie S. Lv D. Zhang Y. Deng J. Zeng L. (2016). A reduction in the butyrate producing species Roseburia spp. and Faecalibacterium prausnitzii is associated with chronic kidney disease progression. Antonie Van Leeuwenhoek. 109 (10), 1389–196. 10.1007/s10482-016-0737-y 27431681

[B29] Kanemitsu Y. Mishima E. Maekawa M. Matsumoto Y. Mano N. (2019). Comprehensive and semi-quantitative analysis of carboxyl-containing metabolites related to gut microbiota on chronic kidney disease using 2-picolylamine isotopic labeling LC-MS/MS. Sci. Rep. 9 (1), 19075. 10.1038/s41598-019-55600-1 31836785 PMC6910927

[B30] Kelly C. J. Zheng L. Campbell E. L. Saeedi B. Scholz C. C. Bayless A. J. (2015). Crosstalk between Microbiota-Derived Short-Chain Fatty Acids and Intestinal Epithelial HIF Augments Tissue Barrier Function. Cell Host Microbe 17 (5), 662–671. 10.1016/j.chom.2015.03.005 25865369 PMC4433427

[B31] Kikuchi K. Saigusa D. Kanemitsu Y. Matsumoto Y. Thanai P. Suzuki N. (2019). Gut microbiome-derived phenyl sulfate contributes to albuminuria in diabetic kidney disease. Nat. Commun. 2019 10 (1), 1835. 10.1038/s41467-019-09735-4 PMC6478834 31015435

[B32] Konrad L. Andersen K. Kesper M. S. Kumar S. V. Anders H.-J. (2019). The gut flora modulates intestinal barrier integrity but not progression of chronic kidney disease in hyperoxaluria-related nephrocalcinosis. Nephrol. Dial Transpl. 35 (1), 86–97. 10.1093/ndt/gfz080 31081025

[B33] Lau W. L. Kalantar-Zadeh K. Vaziri N. D. (2015). The Gut as a Source of Inflammation in Chronic Kidney Disease. Nephron 130 (2), 92–98. 10.1159/000381990 25967288 PMC4485546

[B34] Li Y. Su X. Zhang L. Liu Y. Shi M. Lv C. (2015). Dysbiosis of the gut microbiome is associated with CKD5 and correlated with clinical indices of the disease: a case–controlled study. J. Transl. Med. 17 (1), 228. 10.1186/s12967-019-1969-1 PMC6637476 31315634

[B35] Liu W. C. Tomino Y. Lu K. C. (2018). Impacts of Indoxyl Sulfate and p-Cresol Sulfate on Chronic Kidney Disease and Mitigating Effects of AST-120. Toxins 10 (9), 367. 10.3390/toxins10090367 PMC6162782 30208594

[B36] Lu Z. Zeng Y. Lu F. Liu X. Zou C. (2015). Rhubarb Enema Attenuates Renal Tubulointerstitial Fibrosis in 5/6 Nephrectomized Rats by Alleviating Indoxyl Sulfate Overload. PLoS One 10 (12), e0144726. 10.1145/2818302 26671452 PMC4684395

[B37] Luo X. Chen J. Zou C. (2015). The application and dosage of rhubarb in nephropathy. Chin. J. Integr. Tradit. Chin. Western Med. Nephropathy 16 (10), 930–932.

[B38] Luca M. Venanzio V. Giuseppe L. T. Massimo M. Giovanni C. Riccardo R. (2009). Increased intestinal permeability and tight junction alterations in nonalcoholic fatty liver disease. Hepatology 49 (6), 1877–1887. 10.1002/hep.22848 19291785

[B39] Manga T. Schima W. Maier A. Schober E. Mueller-Mang C. Weber M. (2009). Comparison of axial, coronal, and primary 3D review in MDCT colonography for the detection of small polyps: A phantom study. Eur. J. Radiol. 70 (1), 86–93. 10.1016/j.ejrad.2007.11.040 18221849

[B40] Martin-Venegas R. Brufau M. T. Guerrero-Zamora A. M. Mercier Y. Geraert P. A. Ferrer R. (2013). The methionine precursor DL-2-hydroxy-(4-methylthio)butanoic acid protects intestinal epithelial barrier function. Food Chem. 141 (3), 1702–1709. 23870881 10.1016/j.foodchem.2013.04.081

[B41] Matsuda H. Kageura T. Morikawa T. Toguchida I. Harima S. Yoshikawa M. (2000). Effects of stilbene constituents from rhubarb on nitric oxide production in Lipopolysaccharide-Activated Macrophages. Bioorg. Med. Chem. Lett. 10 (4), 323–327. 10.1016/S0960-894X(99)00702-7 10714491

[B42] Metidji A. Omenetti S. Crotta S. Li Y. Nye E. Ross E. (2018). The Environmental Sensor AHR Protects from Inflammatory Damage by Maintaining Intestinal Stem Cell Homeostasis and Barrier Integrity. Immunity 49 (2), 353–362.e5. 10.1016/j.immuni.2018.07.010 PMC6104739 30119997

[B43] Mcclellan W. M. Flanders W. D. (2003). Risk factors for progressive chronic kidney disease. J. Am. Soc. Nephrol. 14 (2), 65–70. 10.1097/01.ASN.0000070147.10399.9E 12819305

[B44] Meijers B. Farré R. Dejongh S. Vicario M. Evenepoel P. (2018). Intestinal Barrier Function in Chronic Kidney Disease. Toxins 10 (7), 298. 10.3390/toxins10070298 PMC6071212 30029474

[B45] Michael C. Karen L. Wen Z. (2012). Irritable bowel syndrome: methods, mechanisms, and pathophysiology. The confluence of increased permeability, inflammation, and pain in irritable bowel syndrome. Am. J. Physiol. Gastrointest. Liver Physiol. 303 (7), 775–785. 10.1152/ajpgi.00155.2012 22837345

[B46] Neyrinck A. M. Etxeberria U. Taminiau B. Daube G. Van Hul M. Everard A. (2017). Rhubarb extract prevents hepatic inflammation induced by acute alcohol intake, an effect related to the modulation of the gut microbiota. Mol. Nutr. Food Res. 61 (1). 10.1002/mnfr.201500899 26990039

[B47] Popolo A. Adesso S. Pinto A. Autore G. Marzocco S. (2014). L-Arginine and its metabolites in kidney and cardiovascular disease. Amino Acids 46 (10), 2271–2286. 10.1007/s00726-014-1825-9 25161088

[B48] Qian Q. (2017). Inflammation: A Key Contributor to the Genesis and Progression of Chronic Kidney Disease. Contrib. Nephrol. 191, 72–83. 10.1159/000479257 28910792

[B49] Ramezani A. Raj D. S. (2014). The gut microbiome, kidney disease, and targeted interventions. J. Am. Soc. Nephrol. 25 (4), 657–670. 10.1681/ASN.2013080905 24231662 PMC3968507

[B50] Ramezani A. Massy Z. A. Meijers B. Evenepoel P. Vanholder R. Raj D. S. (2016). Role of the Gut Microbiome in Uremia: A Potential Therapeutic Target. Am. J. Kidney Dis. 67 (3), 483–498. 10.1053/j.ajkd.2015.09.027 26590448 PMC5408507

[B51] Salim S. Y. Söderholm J. D. (2015). Importance of disrupted intestinal barrier in inflammatory bowel diseases. Inflamm. Bowel Dis. 17 (1), 362–381. 10.1002/ibd.21403 20725949

[B52] Stubbs J. R. House J. A. Ocque A. J. Zhang S. Johnson C. Kimber C. (2016). Serum Trimethylamine-N-Oxide is Elevated in CKD and Correlates with Coronary Atherosclerosis Burden. J. Am. Soc. Nephrol. 27 (1), 305–313. 10.1681/ASN.2014111063 26229137 PMC4696571

[B53] Sun P. P. Perianayagam M. C. Jaber B. L. (2009). Endotoxin-binding Affinity of Sevelamer: A Potential Novel Anti-Inflammatory Mechanism. Kidney Int. Suppl. (114), S20–S25. 10.1038/ki.2009.403 19946323

[B54] Sun H. Zhu C. Zhang H. Wang Y. Luo G. Hu P. (2009). Analysis and Material Basis Comparison of Rhubarb and Its Processed Products. Zhong Cheng Yao. 31 (3), 420–424.

[B55] Taal M. W. Brenner B. M. (2006). Predicting initiation and progression of chronic kidney disease: Developing renal risk scores. Kidney Int. 70 (10), 1694–1705. 10.1038/sj.ki.5001794 16969387

[B56] Tadao A. Yasushi A. Satoshi M. Takafumi W. Yoshihiro O. Shunichi F. (2009). Effect of a carbonaceous oral adsorbent on the progression of CKD: a multicenter, randomized, controlled trial. Am. J. Kidney Dis. 54 (3), 459–467. 10.1053/j.ajkd.2009.05.011 19615804

[B57] Tremaroli V. Backhed F. (2012). Functional interactions between the gut microbiota and host metabolism. Nature 489 (7415), 242–249. 10.1038/nature11552 22972297

[B58] Vaziri N. D. Yuan J. Rahimi A. Ni Z. Said H. Subramanian V. S. (2012). Disintegration of colonic epithelial tight junction in uremia: a likely cause of CKD-associated inflammation. Nephrol. Dial Transpl. 27 (7), 2686–2693. 10.1093/ndt/gfr624 PMC3616758 22131233

[B59] Vaziri N. D. Zhao Y. Y. Pahl M. V. (2016). Altered intestinal microbial flora and impaired epithelial barrier structure and function in CKD: the nature, mechanisms, consequences and potential treatment. Nephrol. Dial Transpl. 31 (5), 737–746. 10.1093/ndt/gfv095 25883197

[B60] Walker A. W. Lawley T. D. (2013). Therapeutic modulation of intestinal dysbiosis. Pharmacol. Res. 69 (1), 75–86. 10.1016/j.phrs.2012.09.008 23017673

[B61] Wang L.-X. Liu T. Hui L.-Q. Li R.-R. Wu H.-W. Liang Y.-H. (2020). Study on antipyretic effect of rhubarb on rats and its antipyretic ingredients. China J. Chin. Materia Medica. 45 (05), 1128–1134. 10.19540/j.cnki.cjcmm.20191226.201 32237456

[B62] Wang J. B. Kong W. J. Wang H. J. (2011). Toxic effects caused by rhubarb are reversed on immature and aged rats. J. Ethnopharmacol. 134 (2), 216–220. 10.1016/j.jep.2010.12.008 21163343

[B63] Wang H. Zhang W. Zuo L. Dong J. Zhu W. Li Y. (2014). Intestinal dysbacteriosis contributes to decreased intestinal mucosal barrier function and increased bacterial translocation. Lett. Appl. Microbiol. 58 (4), 384–392. 10.1111/lam.12201 24354719

[B64] Wang Y. Zhao H. Wang J. Yanling Z. Xiaohe X. (2014). The relationship between the dose and toxicity/efficacy of prepared Rhubarb based on the theory of “you gu wu yun” on the liver. Chin. J. Tradit. Chin. Med. 39 (15), 2918–2923. 25423833

[B65] Wang L. Cui Y. L. Zhang Z. Lin Z. F. Chen D. C. (2017). Rhubarb Monomers Protect Intestinal Mucosal Barrier in Sepsis via Junction Proteins. Chin Med. J. 130 (10), 1218–1225. 28485323 10.4103/0366-6999.205855 PMC5443029

[B66] Wei X. Yang Z. Rey F. E. Ridaura V. K. Davidson N. O. Gordon J. I. (2012). Fatty Acid Synthase Modulates Intestinal Barrier Function through Palmitoylation of Mucin 2. Cell Host Microbe 11 (2), 140–152. 10.1016/j.chom.2011.12.006 22341463 PMC3285413

[B67] Wong J. DeSantis T. Z. Pahl M. Andersen G. L. (2013). A mouse model of pathological small intestinal epithelial cell apoptosis and shedding induced by systemic administration of lipopolysaccharide. Dis. Model Mech. 6 (6), 1388–1399. 10.1242/dmm.013284 24046352 PMC3820262

[B68] Wong J. DeSantis T. Z. Pahl M. Andersen G. L. (2014). Expansion of urease- and uricase-containing, indole- and p-cresol-forming and contraction of short-chain fatty acid-producing intestinal microbiota in ESRD. Am. J. Nephrol. 39 (3), 230–237. 10.1159/000360010 24643131 PMC4049264

[B69] Xiong Y. Chen L. Fan L. Wang L. Zhou Y. Qin D. (2018). Free Total Rhubarb Anthraquinones Protect Intestinal Injury via Regulation of the Intestinal Immune Response in a Rat Model of Severe Acute Pancreatitis. Front. Pharmacol. 9:75. 10.3389/fphar.2018.00075 29487524 PMC5816759

[B70] Yan Y.L. Ha C. W. Y. Campbell C. R. Mitchell A. J. Anuwat D. Jan O. (2012). Increased gut permeability and microbiota change associate with mesenteric fat inflammation and metabolic dysfunction in diet-induced obese mice. PLoS One 2012, 7 (3), e34233. 10.1371/journal.pone.0034233 PMC3311621 22457829

[B71] Yang X. W. Zhao J. Zhang Y. (1998). Rhubarb research: A new malonyl anthraquinone glycoside compound in rhubarb of Qinling Mountains. Zhong Cao Yao 29 (5), 289.

[B72] Yang J. Yoon L. S. Sook K. Y. Young L. H. Won O. S. Gyu K. M. (2018). Intestinal barrier disruption and dysregulated mucosal immunity contribute to kidney fibrosis in chronic kidney disease. Nephrol. Dial Transpl. 34 (3), 419–428. 10.1093/ndt/gfy172 29939312

[B73] Zeng L.-n. Ma Z.-j. Zhao Y.-l. Zhang L.-d. Li R.-s. Wang J.-b. (2013). The protective and toxic effects of rhubarb tannins and anthraquinones in treating hexavalent chromium-injured rats: The Yin/Yang actions of rhubarb. J. Hazard Mater. 246-247, 1–9.V. 10.1016/j.jhazmat.2012.12.004 23276788

[B74] Zeng Y. Q. Dai Z. Lu F. Lu Z. Liu X. Chen C. (2016). Emodin via colonic irrigation modulates gut microbiota and reduces uremic toxins in rats with chronic kidney disease. Oncotarget 7 (14), 17468–17478. 10.18632/oncotarget.8160 27003359 PMC4951226

[B75] Zhang Z. H. Wei F. Vaziri N. D. Cheng X. L. Bai X. Lin R. C. (2015). Metabolomics insights into chronic kidney disease and modulatory effect of rhubarb against tubulointerstitial fibrosis. Sci. Rep. 5, 14472. 10.1038/srep14472 26412413 PMC4585987

[B76] Zhang Z. H. Li M. H. Liu D. Chen H. Chen D. Q. Tan N. H. (2018). Rhubarb Protect Against Tubulointerstitial Fibrosis by Inhibiting TGF-beta/Smad Pathway and Improving Abnormal Metabolome in Chronic Kidney Disease. Front. Pharmacol. 9:1029. 10.3389/fphar.2018.01029 30271345 PMC6146043

[B77] Zhao Q. Chen Y.-p. Cui X.-s. Tian Q.-c. Shen S. Bi D. (2018). Study on multi-compound determination and fingerprint of Rheum palmatum by UPLC. Chin J. Pharm. Anal. 38 (10), 1697–1614.

[B78] Zheng Q.-x. Wu H.-f. Guo J. Nan H.-j. Chen S.-l. Yang J.-s. (2013). Review of Rhubarbs: Chemistry and Pharmacology. Chin. Herb. Med. 5 (1), 9–32. 10.7501/j.issn.1674-6384.2013.01.003

[B79] Zhu Y. M. (2004). Anthraquinone laxative and colorectal melanosis. Chin. J. Digestion 24 (5), 58–59.

