# Supplementary figures and images for: Rhubarb Enema Improved Colon Mucosal Barrier Injury in 5/6 Nephrectomy Rats May Associate With Gut Microbiota Modification
Source: Front Pharmacol. 2020 Jul 29;11:1092. doi: 10.3389/fphar.2020.01092 (PMC7403201; doi:10.3389/fphar.2020.01092)

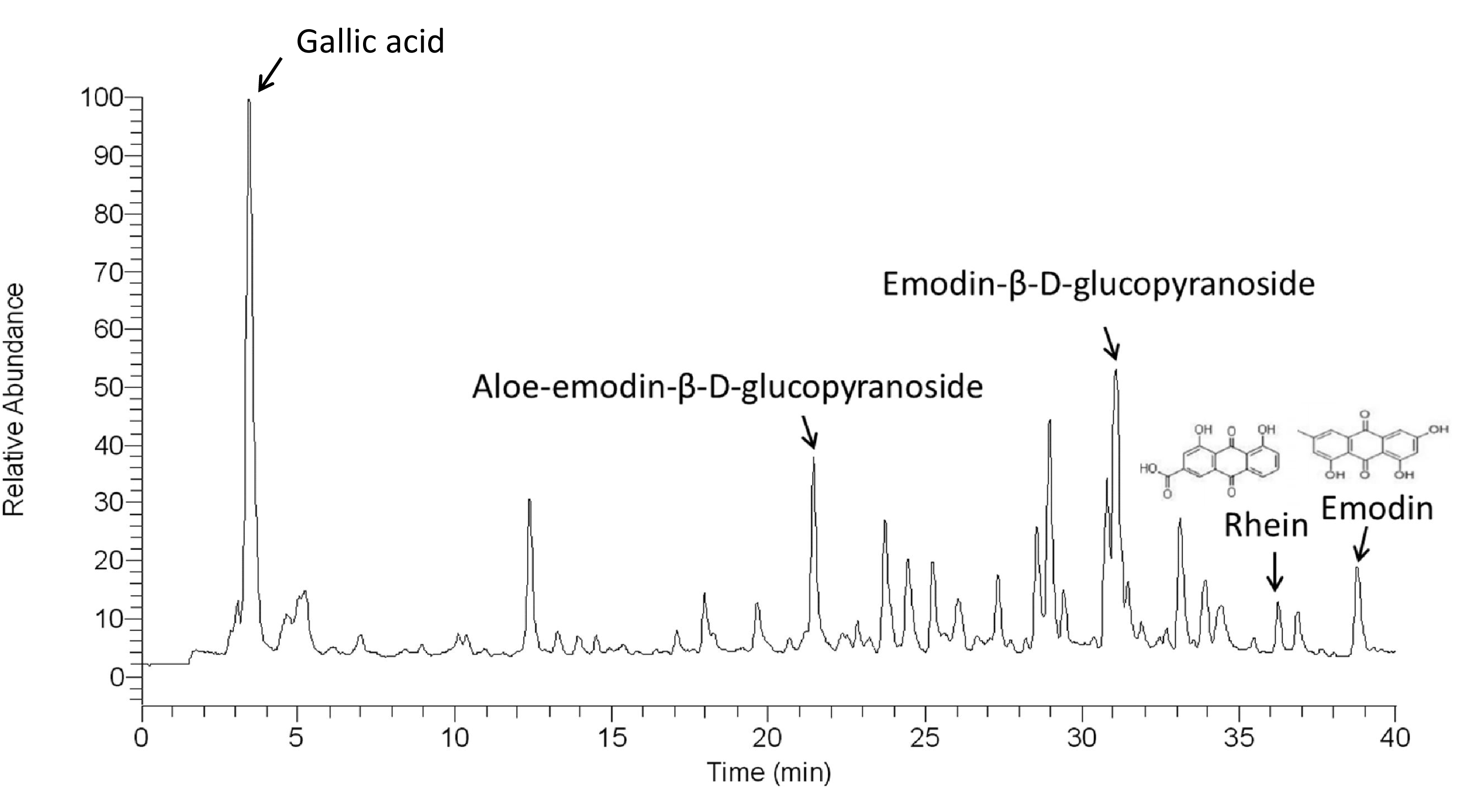

Supplement: Supplementary file 1 [file Image_1.tif]
