# Supplementary material for: Rhubarb Enema Improved Colon Mucosal Barrier Injury in 5/6 Nephrectomy Rats May Associate With Gut Microbiota Modification
Source: Front Pharmacol. 2020 Jul 29;11:1092. doi: 10.3389/fphar.2020.01092 (PMC7403201; doi:10.3389/fphar.2020.01092)
Supplement: Supplementary file 2 [file Table_1.docx]

Chemical constituents from the roots and rhizomes of rhubarb analyzed by UPLC-ESI-MS

| No. | t_R_/min | [M-H]^-^(m/z) | MS^2^  (m/z) | Inferred and compound |
| --- | --- | --- | --- | --- |
| 1 | 3.46 | 169.0139 | 125.0448 | Gallic acid (C_7_H_6_O_5_) |
| 2 | 21.64 | 431.0971 | 169.1101, 125.3890 | Aloe-emodin-β-D-glucopyranoside (C_21_H_20_O_10_) |
| 3 | 31.08 | 431.0968 | 269.0461, 257.0461, 239.0719 | Emodin-β-D-glucopyranoside (C_21_H_20_O_10_) |
| 4 | 36.21 | 283.0228 | 239.0326, 211.0367, 183.0422 | Rhein  (C_15_H_8_O_6_) |
| 5 | 38.85 | 269.0465 | 241.0477, 225.0532, 197.0576 | Emodin (C_15_H_10_O_5_) |
